# Risk-managed antidepressant prescribing in primary care mental health: what pharmacogenetics can (and cannot) deliver

**DOI:** 10.3389/fmed.2026.1814980

**Published:** 2026-04-15

**Authors:** Waseem Jerjes, Daniel Harding, Marcin Klingbajl

**Affiliations:** 1 Department of Primary Care and Public Health, Faculty of Medicine, Imperial College London, London, United Kingdom; 2 North End Medical Centre, Hammersmith and Fulham Primary Care Network, London, United Kingdom

**Keywords:** adverse drug reactions, antidepressants, clinical decision-making, depression, pharmacogenetics, precision prescribing, primary care, treatment-resistant depression

## Introduction

Most mental health care happens in primary care, where antidepressants are prescribed at scale and often under time pressure. Yet the dominant prescribing model remains incremental “trial-and-error”: start a first-line antidepressant, monitor tolerability and symptoms, then switch or augment if response is inadequate. For many patients this is acceptable; for others, early adverse effects, drug–drug interactions, and repeated non-response compound distress, functional impairment, and disengagement from care. The clinical reality is that primary care needs tools that reduce avoidable prescribing harm while preserving simplicity, safety, and equity.

Pharmacogenetics (PGx), testing genetic variants that influence drug metabolism and, less consistently, drug targets, offers a plausible route to move from trial-and-error toward risk-managed prescribing. Importantly, PGx does not “match” a patient to a perfect antidepressant, and it does not replace shared decision-making, psychosocial care, or measurement-based follow-up. What it can do, when used well, is identify patients at higher risk of clinically meaningful drug–gene interactions (e.g., altered CYP2D6/CYP2C19 metabolism) and support dose selection or medication choice to reduce that risk (1, 2). This opinion paper argues for a pragmatic, primary-care-ready model: use PGx as a safety and risk-stratification tool, deployed at high-yield decision points, rather than as a promise of precision “cure.”

To inform this opinion paper, we performed a structured narrative search of PubMed on 24 March 2026 using combinations of the terms “pharmacogenetics,” “pharmacogenomics,” “antidepressants,” “depression,” “major depressive disorder,” “primary care,” “CYP2D6,” “CYP2C19,” “CYP2B6,” “sertraline,” “SLC6A4,” “HTR2A,” “implementation,” and “cost-effectiveness.” We prioritized English-language guidelines, randomized trials, systematic reviews/meta-analyses, implementation studies, and health technology assessments relevant to adult antidepressant prescribing, and we hand-searched reference lists of key papers to identify additional relevant sources. Conference abstracts, non-peer-reviewed material, and studies without clear clinical or implementation relevance to primary care were not prioritized.

## The evidence base is real—but modest, heterogeneous, and easy to oversell

Randomized trials and meta-analyses now provide a clearer picture of what PGx testing delivers in depression. In the pragmatic PRIME Care trial (*n* ≈ 1,944), provision of commercial PGx results reduced prescriptions with predicted drug–gene interactions; although the original 2022 analysis showed only modest absolute differences in remission, a prespecified 2025 *post hoc* time-to-event analysis found faster remission and response in the PGx-guided arm, with benefits that persisted across the full 24-week follow-up (3, 4). Other controlled trials, including the Canadian patient- and rater-blinded GAPP-MDD trial and a trial in older adults, have reported improvements in symptom outcomes or trends in that direction, though effect sizes vary and some studies remain underpowered or context-specific (5, 6). A more recent single-center RCT reported higher remission and response rates and fewer adverse reactions with PGx-guided care, but the lack of clinician blinding and single-center design remain important caveats for generalisability to routine primary care (7).

When pooled, systematic reviews and meta-analyses generally suggest that PGx-guided prescribing is associated with a modest improvement in remission or response compared with unguided care, but with substantial heterogeneity across tests, populations, outcomes, and follow-up windows (8–14). This now includes newer 2025 analyses: a cumulative meta-analysis of randomized trials found moderate benefits at 8 weeks but less certain remission benefit by 12 weeks, and a separate weighted multigene meta-analysis reported a higher likelihood of remission compared with unguided care (13, 14). The more “positive” pooled estimates commonly arise from a mixture of open-label and blinded designs, or from specific commercial tools with differing algorithms, gene content, and reporting formats. This heterogeneity matters in primary care: it limits the extent to which any one trial or panel can be assumed to apply to a local setting, and it makes “PGx works/doesn't work” an unhelpful binary (15–18).

Two additional realities deserve emphasis. First, the strongest and most consistently actionable PGx signals in antidepressant prescribing are pharmacokinetic, particularly CYP2D6, CYP2C19, and, for some agents, CYP2B6 (1, 2). By contrast, pharmacodynamic genes commonly included in commercial panels—especially SLC6A4 and HTR2A—do not have sufficiently consistent, guideline-grade evidence to support routine antidepressant selection or dosing decisions (1, 19). In practical terms, these variants do not reliably predict which SSRI will work for an individual patient, so their inclusion in panel reports can create a misleading impression of precision if they are presented as decisive treatment markers. Other candidate genes, such as ABCB1, show at best limited or inconsistent associations with clinical outcomes. Second, the clinical utility of combinatorial panels depends not only on genotype, but on how results are translated into phenotype and then into transparent, clinically coherent recommendations—something that still varies across products and implementation settings (15–23).

The key point is not to wait for perfect evidence, but to be clear about what PGx can realistically deliver in primary care. At present, its strongest case is as a tool to reduce avoidable harm and shorten the time to a tolerable, guideline-concordant regimen—particularly for patients at higher risk of medication problems—rather than as a universal solution for all depression.

## High-yield clinical scenarios for primary care

Primary care can operationalise PGx without turning every antidepressant start into a genomic event. A risk-managed approach focuses on situations where the probability and consequences of a drug–gene interaction are higher, where the alternative is repeated switching, or where adverse effects carry disproportionate burden.

### After a problematic first trial: intolerance, early discontinuation, or “multiple switches”

The most practical trigger is not “diagnosis of depression” but “a decision point that would otherwise lead to another switch.” When a patient stops a first antidepressant because of activation, nausea, sedation, sexual dysfunction, or other early adverse effects—and when adherence becomes fragile—PGx can add value by reducing the chance that the next trial repeats an avoidable exposure. In this setting, a CYP2D6 or CYP2C19 poor or ultrarapid metaboliser phenotype can plausibly shift a clinician toward a lower starting dose, slower titration, or an alternative agent with less dependence on the implicated pathway, consistent with CPIC and DPWG guidance (1, 2). In short: PGx is most useful when the clinical problem is “tolerability and risk,” not “predicting a perfect response.”

### Polypharmacy, multimorbidity, and older adults

Primary care patients frequently take multiple medications, and antidepressants are commonly co-prescribed with drugs that inhibit or induce CYP enzymes (e.g., some PPIs, antifungals, antiarrhythmics) or that add anticholinergic/sedative burden. In older adults, physiological vulnerability, falls risk, and the clinical cost of adverse effects are amplified. A randomized trial in older adults found improved outcomes with combinatorial PGx-informed selection compared with treatment as usual (6). While one trial does not define policy, it supports the intuitive notion that where medication harm is costlier, risk-stratification tools have higher yield. In these contexts, PGx should be interpreted alongside drug–drug interactions and organ function (renal/hepatic), not as a standalone “green-yellow-red” directive (17, 18).

### When primary care manages anxiety, mixed presentations, and rapid cycling between medications

Primary care rarely sees “textbook” major depression. Symptoms overlap with anxiety, insomnia, chronic pain, and perimenopausal or inflammatory states. The result is frequent medication cycling, including short trials, dose changes, and combinations. A recent systematic review and meta-analysis addressing drug-metabolizing enzyme variation across mood and anxiety disorders highlights that pharmacokinetic variation is relevant across common primary-care mental health presentations (12). In these messy real-world phenotypes, PGx cannot resolve diagnostic uncertainty, but it can reduce one avoidable layer of prescribing risk when clinicians need to move between agents.

### Setting expectations: how to talk about PGx with patients

Implementation work consistently shows that expectations and communication determine whether PGx supports or undermines therapeutic alliance. A scoping review of patient and public attitudes found enthusiasm for PGx but also concerns about privacy, misunderstanding of what results mean, and uncertainty about how results will be used (24). Qualitative work involving people with lived experience of depression and clinicians/policy stakeholders highlights the need for clear explanation of benefits, limitations, and safeguards (25). Primary care counseling can be brief and accurate: “This test cannot tell us which antidepressant will work best for you, but it can flag medicines that your body may process unusually fast or slow, which can affect side effects and dosing.”

## A primary-care pathway: make PGx simple, transparent, and shared

If PGx is to change practice rather than add noise, primary care needs a pathway that answers four questions: Who should be tested? What should be tested? Who interprets results? How are results stored and reused?

### Who to test: targeted, not universal

Health technology assessments and reviews note that clinical utility and cost-effectiveness depend heavily on patient selection, local costs, and the clinical alternative (26–29). A targeted approach is therefore more defensible than universal pre-emptive testing. Reasonable triggers include: (i) one antidepressant stopped due to significant adverse effects; (ii) two inadequate trials or multiple switches; (iii) complex polypharmacy with known CYP inhibitors/inducers; (iv) older adults or medically frail patients where adverse effects have high downstream consequences; and (v) a history suggesting atypical sensitivity or non-response. These triggers align with a risk-management logic rather than a “genotype everyone” logic.

### What to test: prioritize actionable pharmacokinetics and guideline-anchored outputs

Primary care should privilege test outputs that map to guideline-anchored actions. CPIC provides recommendations for using CYP2D6, CYP2C19, and CYP2B6 results to inform serotonin reuptake inhibitor prescribing, and explicitly states that current evidence does not support routine clinical use of SLC6A4 and HTR2A for these decisions (1). DPWG similarly provides actionable recommendations for SSRIs with CYP2D6/CYP2C19 (2). This points to an important procurement principle: whatever panel is used, its key outputs should include robust allele calling and phenotype translation, plus a clear path to CPIC/DPWG-consistent recommendations rather than opaque composite scores.

Sertraline is a particularly relevant primary-care example because CPIC now treats it as a combined CYP2C19/CYP2B6 question when both results are available. CYP2C19 poor metabolisers may warrant a lower starting dose, slower titration, and around 50% lower maintenance dosing or an alternative antidepressant, while CYP2B6 poor metabolisers may also require a lower starting dose, slower titration, and around 25% lower maintenance dosing; when both pathways are unfavorable, the case for dose reduction or an alternative strengthens further (1). For a drug so commonly used in primary care, this is where PGx can genuinely support safer and more transparent prescribing.

### Who interprets: a team-based model with pharmacist stewardship

Primary care cannot absorb complex genomic interpretation without support. Implementation evaluations and systematic reviews highlight barriers including limited clinician time, variable knowledge/confidence, unclear professional roles, and lack of decision support (30, 31). A practical model is pharmacist-supported interpretation (in-practice, PCN, or health-system level), with concise recommendations returned to the GP. In systems where pharmacist prescribing is established, that role may extend beyond interpretation to collaborative antidepressant optimisation within an agreed scope of practice, whereas in other settings pharmacists may remain in an advisory role. Workforce reviews indicate positive attitudes toward PGx but gaps in knowledge and confidence—suggesting that education and role clarity are prerequisites for safe scaling (32). Table 1 summarizes a pragmatic workflow that keeps prescribing responsibility explicit while allowing the pharmacist role to range from interpretive support to pharmacist-prescriber input, depending on local regulation and team design.

**Table 1:** A phase-based pragmatic pharmacogenetics pathway for antidepressant prescribing in primary care.

Step/decision point	What to do in primary care	Minimum PGx outputs to act on	“Non-PGx” checks that must run in parallel	Practical documentation and follow-up
Phase 1. Pre-testing/case selection				
New antidepressant start (low complexity)	Usual evidence-based first-line prescribing; do not default to PGx	None	Baseline PHQ-9/GAD-7; key comorbidities; suicide risk screen; interaction check	Record baseline scores; early review plan (1–2 weeks for tolerability; 4–6 weeks for response)
Stopped first antidepressant due to significant adverse effects	Order PGx at the next switch, or before switch if delay is short; consider interim non-PGx switch	CYP2D6 and CYP2C19 phenotype, and CYP2B6 where relevant	Drug–drug interactions (including CYP inhibitors/inducers), QT risk, hepatic/renal function, alcohol/substance use	Record adverse effects and timing; document PGx counseling (“risk tool, not predictor”)
Two inadequate trials/repeated switching	Use PGx to reduce avoidable re-exposure to the same problem; consider pharmacist-supported review	CYP2D6, CYP2C19, CYP2B6, with clear guideline-anchored recommendations	Confirm adequate dose and duration, assess adherence, review diagnosis, psychosocial stressors	Reset expectations; schedule structured follow-up; consider referral thresholds for complex or treatment-resistant depression
Older adult/frailty/high consequences of side effects	Lower threshold for PGx if starting or switching; prioritize a tolerability-optimized plan	Actionable metabolism phenotypes; avoid opaque “black box” composite scores	Falls-risk medicines, anticholinergic burden, hyponatraemia risk, polypharmacy review	Document harm-avoidance rationale; monitor side effects proactively
15.6-7.4,-39499pt Complex polypharmacy with known CYP inhibitors/inducers	Use PGx together with interaction management to choose dose or agent	Genotype/phenotype plus interaction-aware recommendation	Full medication reconciliation; interaction checker; consider deprescribing	Store phenotype in EHR problem list or alerts (e.g. “CYP2D6 poor metaboliser”)
Phase 2. Analysis/interpretation and prescribing				
15.6-7.4,-51498pt PGx report received	Implement CPIC/DPWG-consistent actions (dose adjustment, avoidance, or alternative choice); if unclear, seek pharmacist or genetics support	Phenotype translation must be explicit; recommendations must be traceable to guidelines	Recheck interactions after final regimen is chosen	Record chosen action and rationale; attach report; plan review of response and tolerability
Phase 3. Post-testing/storage and reuse				
“Test once, use often” maintenance	Reuse PGx for future relevant prescribing, including beyond antidepressants	Stored phenotype in a computable EHR field	Update medication list; reassess if new strong inhibitors or inducers are added	Keep a patient-facing summary line in the record; revisit consent and privacy preferences where relevant

### How to store and reuse results: “test once, use often”

Unlike many investigations, PGx results are potentially lifelong. The implementation goal should therefore be reusability: storing genotype/phenotype (e.g., CYP2D6 poor metaboliser) in the electronic record in a computable form, alongside the report. This reduces repeat testing, supports future prescribing beyond antidepressants, and improves the value proposition. It also allows future reinterpretation as allele function assignments evolve (21, 22).

### Equity, trust, and governance

Equitable implementation is not automatic. Reviews on race, health equity, and pharmacogenomics warn that under-representation of diverse populations and variable allele frequencies can compound disparities if tests, interpretation, and access are not designed with equity in mind (33). In primary care, governance should include: transparent consent language, clear data handling policies, options for patients to decline testing without penalty, and pathways to subsidized testing for patients who meet clinical criteria but face cost barriers. Without these safeguards, PGx risks becoming a “premium” add-on rather than a safety tool.

## Discussion

PGx is ready for pragmatic use in primary care mental health—but only if we stop asking it to do the wrong job. The best supported contribution of PGx in depression care is not a deterministic prediction of antidepressant response; it is risk reduction through identification of clinically meaningful drug–gene interactions and translation into dosing or selection choices anchored in guidelines (1–3). The most consistent signal across the evidence base is a modest improvement in outcomes, with substantial heterogeneity across tools and study designs (8–14). That pattern supports a targeted “risk managed prescribing” model rather than universal testing.

PGx should also be paired with measurement based care (e.g., PHQ 9/GAD 7), early follow up after dose changes, and attention to psychosocial drivers of symptoms; otherwise, a genomic report can distract from the bigger determinants of non-response.

Practice change, therefore, should focus on (i) defining high yield triggers for testing; (ii) privileging actionable pharmacokinetic genes with transparent reporting; (iii) embedding PGx into team-based medication review and shared decision making; and (iv) building EHR and governance infrastructure that supports reuse and equity. The pathway proposed here is intentionally conservative: it aims to reduce avoidable harm and shorten the time to a tolerable regimen for patients who are otherwise likely to experience repeated switching, adverse effects, or medication complexity.

A further implementation risk is that PGx becomes either over authoritative (“the report says no”) or ignored (“too complex to use”), both of which undermine safe prescribing. Primary care needs explicit governance that frames PGx as one input to clinical reasoning, not a substitute for it: results should be documented with a short rationale for the chosen action (dose adjustment, alternative selection, or no change), with clear acknowledgment of uncertainty and the parallel role of drug–drug interactions, comorbidity, and patient preference (1, 2, 17, 19). Governance should also protect equity: patients should not be denied treatment while awaiting testing, nor should PGx become a precondition for access to care; instead, it should be deployed at defined high yield decision points and audited for differential access and outcomes across population groups (33). Done this way, PGx supports defensible, risk managed prescribing rather than creating a new form of “genetic gatekeeping.”

Even strong evidence will not change practice if PGx results are not actionable at the point of prescribing. The practical requirement is simple: “test once, use often” only works if phenotype and key warnings are stored in a reusable, visible form within the electronic record, ideally supported by decision support that surfaces relevant drug–gene and drug–drug interactions during prescribing and medication review (30, 31). That support should be selective rather than noisy: generic or low-priority PGx alerts risk alert fatigue, so systems should prioritize clearly actionable, high-severity drug–gene interactions and suppress low-value warnings wherever possible (34). This is also where pharmacist stewardship is most valuable: implementation studies consistently show that role clarity, workflow design, and confidence in interpretation are more limiting than enthusiasm for the concept (30–32). A realistic primary care model therefore pairs light-touch clinician training (what PGx can and cannot tell us; how to communicate results) with pharmacist-led interpretation and EHR-embedded prompts, so PGx becomes routine safety infrastructure rather than an occasional specialist add-on.

Evidence from the UK suggests that mental health clinical pharmacist independent prescribers can contribute meaningfully within general practice, while recent Slovenian studies indicate that pharmacist prescriber models in primary care may improve clinical outcomes and quality of life and are viewed positively by GPs, pharmacist prescribers, and patients (35–37). However, these models are context dependent: in some countries GPs may be less supportive, and implementation is likely to work best where prescribing authority, governance, training, and collaborative role boundaries are clearly defined rather than assumed.

Several research and implementation gaps remain. Primary care needs independent, head to head comparisons of test panels and reporting formats; clearer evidence on which patient subgroups benefit most; and pragmatic evaluations of pharmacist-supported and pharmacist-prescriber workflows, including impact on workload, equity, patient-reported experience, and prescribing governance (25, 30, 31, 35–37). Because effects are modest and heterogeneous, PGx implementation should be accompanied by pragmatic measurement rather than assumed benefit. Health systems can evaluate impact using outcomes that matter to primary care: early discontinuation due to side effects, time to a tolerated therapeutic dose, number of medication switches/augmentations, burden of adverse effects, and remission/response trajectories using standard measures such as PHQ 9/GAD 7, alongside safety outcomes in higher risk groups (3, 8–14, 19). Process measures are equally important: proportion of eligible patients receiving testing, turnaround time, concordance with guideline anchored actions, and reusability of results across future prescribing (1, 2, 21, 22). Economic evidence is encouraging in some models but sensitive to local assumptions; health systems should evaluate cost effectiveness in their own delivery context rather than importing conclusions wholesale (26–29). Finally, clinicians should remain cautious about expanding PGx decision making into pharmacodynamic genes without guideline grade support (1, 23).

In the meantime, primary care can act now: treat pharmacogenetics as a safety technology. Used selectively, explained clearly, and delivered through a team based pathway, PGx can help primary care move from trial and error toward a more transparent and defensible form of prescribing—one that manages risk, respects patient preferences, and makes better use of the time clinicians already spend switching antidepressants.
